# Telomere length and telomerase activity in malignant lymphomas at diagnosis and relapse

**DOI:** 10.1054/bjoc.1999.0970

**Published:** 2000-01-18

**Authors:** K Remes, K-F Norrback, R Rosenquist, C Mehle, J Lindh, G Roos

**Affiliations:** Departments of Pathology and Oncology, University of Umeå, Umeå, S-90187, Sweden

**Keywords:** telomere length, telomerase activity, malignant lymphoma, relapse, tumour progression, clonality

## Abstract

Telomere length maintenance, in the vast majority of cases executed by telomerase, is a prerequisite for long-term proliferation. Most malignant tumours, including lymphomas, are telomerase-positive and this activity is a potential target for future therapeutic interventions since inhibition of telomerase has been shown to result in telomere shortening and cell death in vitro. One prerequisite for the suitability of anti-telomerase drugs in treating cancer is that tumours exhibit shortened telomeres compared to telomerase-positive stem cells. A scenario is envisioned where the tumour burden is reduced using conventional therapy whereafter remaining tumour cells are treated with telomerase inhibitors. In evaluating the realism of such an approach it is essential to know the effects on telomere status by traditional therapeutic regimens. We have studied the telomere lengths in 47 diagnostic lymphomas and a significant telomere shortening was observed compared to benign lymphoid tissues. In addition, telomere length and telomerase activity were studied in consecutive samples from patients with relapsing non-Hodgkin's lymphomas. Shortened, unchanged and elongated telomere lengths were observed in the relapse samples. The telomere length alterations found in the relapsing lymphomas appeared to be independent of telomerase and rather represented clonal selection random at the telomere length level. These data indicate that anti-telomerase therapy would be suitable in only a fraction of malignant lymphomas. © 2000 Cancer Research Campaign


*British Journal of Cancer* (2000) **82**(3), 601–607
© 2000 Cancer Research Campaign
DOI: 10.1054/ bjoc.1999.0970, available online at http://www.idealibrary.com on IDEAL®

### Telomere length and telomerase activity in malignant lymphomas at diagnosis and relapse

**K Remes[1], K-F Norrback[1], R Rosenquist[1], C Mehle[1], J Lindh[2] and G Roos[1]**

Departments of [1]Pathology and [2]Oncology, University of Umeå, S-90187, Umeå, Sweden

**Summary** Telomere length maintenance, in the vast majority of cases executed by telomerase, is a prerequisite for long-term proliferation. Most malignant tumours, including lymphomas, are telomerase-positive and this activity is a potential target for future therapeutic interventions since inhibition of telomerase has been shown to result in telomere shortening and cell death in vitro. One prerequisite for the suitability of anti-telomerase drugs in treating cancer is that tumours exhibit shortened telomeres compared to telomerase-positive stem cells. A scenario is envisioned where the tumour burden is reduced using conventional therapy whereafter remaining tumour cells are treated with telomerase inhibitors. In evaluating the realism of such an approach it is essential to know the effects on telomere status by traditional therapeutic regimens. We have studied the telomere lengths in 47 diagnostic lymphomas and a significant telomere shortening was observed compared to benign lymphoid tissues. In addition, telomere length and telomerase activity were studied in consecutive samples from patients with relapsing non-Hodgkin's lymphomas. Shortened, unchanged and elongated telomere lengths were observed in the relapse samples. The telomere length alterations found in the relapsing lymphomas appeared to be independent of telomerase and rather represented clonal selection random at the telomere length level. These data indicate that anti-telomerase therapy would be suitable in only a fraction of malignant lymphomas. © 2000 Cancer Research Campaign

**Keywords:** telomere length; telomerase activity; malignant lymphoma; relapse; tumour progression; clonality

In normal somatic cells telomere shortening occurs with each cell division whereas immortalized, permanently growing cell lines demonstrate maintenance of telomeric sequences with stabilization of their telomeric lengths at highly variable levels ranging from a few to >20 kbp in individual cell lines. In order to compensate for the telomere shortening that occurs upon cell division due to the 'end replication problem' a specific activity with the ability to add new telomeric $T_2AG_3$ sequences to the ends of the chromosomes is needed (Levy et al, 1992). In most cases this function is executed by telomerase, a ribonucleoprotein complex with reverse transcriptase activity (Morin, 1989). Telomerase expression is a characteristic feature of permanent cell lines and a vast majority human malignant tumours whereas most normal somatic tissues have been found negative (Kim et al, 1994). Specific somatic cells like activated B and T lymphocytes and certain stem cells also exhibit telomerase activity (Hiyama et al, 1995*b*; Härle-Bachor and Boukamp, 1996; Norrback and Roos, 1997). However, despite the expression of telomerase these cell types have been shown to undergo telomere attrition both in vivo and in vitro although possibly at a decreased rate (Vaziri et al, 1994; Weng et al, 1995; Norrback and Roos, 1997). The only exception appears to be germinal centre B-cells which have been reported to exhibit telomere elongation (Weng et al, 1997). A fraction of in vitro immortalized human cells have been reported to exhibit alternative telomere length maintenance mechanisms independent of telomerase (ALT), but in vivo such a phenotype, characterized by extremely long and heterogenous telomeres, seems to be rare (Mehle et al, 1996; Bryan and Reddel, 1997; Bryan et al, 1997).

In order for normal cells, lacking telomerase activity, to become immortal it has been proposed, based on in vitro studies, that two proliferative lifespan barriers, mortality stage M1 and M2, have to be overcome. After a certain number of cell divisions the telomeres would have shortened to a minimum length and this shortening will signal to the cells to enter M1, i.e. senescence. The senescent cell is characterized by cell cycle arrest, resistance to undergo apoptosis and can remain viable for a long period of time. Depending on the specific cell-type, functional p53 and/or Rb proteins are essential for entry into senescence and inactivation of these tumour suppressors will allow the cells to escape the M1-barrier and undergo further rounds of cell division (Wynford-Thomas, 1997). These cells will acquire critically shortened telomeres resulting in a proliferative crisis with massive cell death. Rare cells which have reactivated telomerase will escape the M2-barrier and become immortal.

The expression level of hTERT, the catalytic subunit of the telomerase complex, seems to be present in limiting amounts and is strongly correlated to the level of telomerase activity (Meyerson et al, 1997). Introduction of hTERT into normal cells lacking telomerase has been found to give a prolonged lifespan, far beyond that of normal cells, proving the linkage between telomere length and replicative lifespan (Bodnar at al, 1998; Morales et al, 1999). In these cells the senescence barrier is circumvented but is still intact and the cells can be induced to senesce if the telomerase expression is turned off with subsequent telomere shortening.

Since telomerase is strongly associated with malignancy it has been identified as a potential target for anti-tumour therapy. A number of anti-telomerase treatment approaches, such as anti-sense oligonucleotides and reverse transcriptase inhibitors have

*Received 6 May 1999*
*Revised 2 September 1999*
*Accepted 9 September 1999*

*Correspondence to:* K-F Norrback been successful in inducing telomere erosion and cell death in different permanent cell lines (Feng et al, 1995; Strahl and Blackburn, 1996). Also, other ways of inhibiting the action of telomerase are emerging like DNA tetraplex interacting agents (Jean-Louis and Claude, 1998). A scenario is thereby envisioned where the tumour mass is reduced in size by surgery or chemotherapy whereafter inhibition of telomerase is expected to result in progressive telomere shortening and induction of senescence or cell death in the remaining tumour cells before a potential relapse would appear. Whether the effect of the anti-telomerase drugs will be induction of senescence or crisis and cell death depends on whether the tumour cells possess an intact senescence programme.

For two reasons fairly short telomeres in the tumour cells to be treated is a prerequisite for a successful clinical outcome using anti-telomerase treatment strategies. Firstly, only tumour cells with short telomeres could be induced to senesce or undergo cell death after a limited number of cell divisions. Secondly, anti-telomerase drugs will lead to telomere shortening of the telomerase-positive stem cells as well and the drugs must be effective before severely damaging normal stem cells. Comparisons of normal and tumour tissues have generally demonstrated shorter telomeres in the tumour samples, but some tumours have demonstrated similar or extended telomere lengths (Hiyama et al, 1995*a*; Mehle et al, 1996; Norrback and Roos, 1997; Bryan et al, 1997). Thus, telomere length estimations should be performed on the tumour tissue before anti-telomerase treatment is instituted in order to select cases suitable for this type of therapy.

So far no studies have been performed to assess the role and suitability of anti-telomerase drugs in the clinical setting of tumour therapy. By analysing the telomere length status of lymphomas at diagnosis and at subsequent relapses, predictions can be made concerning the suitability of anti-telomerase therapy. A simple interpretation would be that for patients with short telomeres in the relapse tumour material anti-telomerase treatment could have been beneficial, whereas this is less likely for cases with intermediate or long telomeres. We decided to analyse this in a series of diagnostic malignant lymphomas, including 16 relapse cases, which were analysed with respect to telomere length. Considerable individual differences concerning telomere peak lengths of the lymphomas were observed whereas the mean telomere length values showed less variation. At relapse constant, shortened or lengthened telomeres were found. These data implicate that anti-telomerase therapy will be effective in only a fraction of malignant lymphomas.

### MATERIALS AND METHODS

### Samples

Tumour DNA was isolated at diagnosis from 47 malignant lymphomas, nine samples from benign lymphoid tissues and from two to five samples from 16 patients with relapsing B- or T-cell lymphomas. From 14 out of the 16 relapsing lymphomas diagnostic samples were available and were thus included in the group with diagnostic lymphomas. The samples were mainly from lymph nodes but also material from bone marrow, peripheral blood and pleural effusions were obtained. All cases were characterized by immunophenotyping using flow cytometry and a panel of monoclonal antibodies directed against various surface antigens. Based on the immunophenotype the fraction of malignant cells could be estimated in each sample. The lymphomas were classified according to the REAL classification (Harris et al, 1994). At diagnosis, 22 cases were small cell lymphomas (12 follicular follicle centre cell lymphomas, five chronic lymphocytic leukaemias, four lymphoplasmacytoid lymphomas and one mantle cell lymphoma) and 19 were large cell lymphomas. Six cases had Hodgkin's disease. Morphologic diagnoses for the 16 relapsing lymphomas are given in Table 2.

### Telomere length measurements

Southern blotting, hybridization using a telomeric probe and calculations of mean telomere length and peak telomere length were performed as described (Mehle et al, 1996). Briefly, 10 μg of restriction enzyme-digested genomic DNA of a sample were loaded onto 0.7% agarose gels. After Southern transfer the telomeric fragments were detected through hybridization with the telomeric probe $(TTAGGG)_4$. The autoradiographs were scanned (Personal Densitometer, Molecular Dynamics, USA) and the mean telomere length of a sample was calculated over the range of 2–23 kbp. Telomere peak lengths were measured by estimating the band size corresponding to the point with the highest optical density within the peak. In order to check for adequate DNA fragmentation by the restriction enzymes and to normalize for differences in DNA loading filters were hybridized with a microsatellite probe, $(CAC)_5$ as described (Mehle et al, 1996).

### Telomerase activity measurements

Protein measurements and preparation of extracts were performed as described (Norrback et al, 1996). All extracts were diluted to 0.14 μg protein $\mu l^{-1}$ and were analysed at 0.28 μg $assay^{-1}$, which is within the linear range of the assay, as described (Wright et al, 1995; Holt et al, 1996; Norrback et al, 1998). The polymerase chain reaction (PCR)-based Telomeric Repeat Amplification Protocol (TRAP) was made semi-quantitative with inclusion of the Internal Telomerase Assay Standard (ITAS) used at 15 attograms $assay^{-1}$ as described (Wright et al, 1995; Norrback et al, 1998). The telomerase activity value is the ratio between the amplified telomerase products and the amplified ITAS product. The telomerase activity value of a sample is the mean of two or more analyses. Telomerase positive cell lines and RNAse incubation of selected samples to prove the RNA-template dependency of telomerase in the formation of telomeric products were controls used in the study in addition to the internal standard.

### RESULTS

The 47 diagnostic lymphoma samples demonstrated similar mean telomere lengths in the different diagnostic subgroups with mean lengths varying from 5.1 to 5.4 kbp (Table 1). For the benign lymphoid tissues this figure was 7.9 kbp. All the separate lymphoma subgroups exhibited significantly shortened telomeres compared to the benign samples ($P < 0.01$ using Mann–Whitney). All the benign samples exhibited one peak and the length of the peaks ranged from 6.9 to 12.6 kbp. A more heterogenous picture emerged concerning peak lengths of the lymphomas which exhibited one or multiple peaks that ranged in length from 2.4 to >23 kbp and considerable individual differences were observed (Tables 1 and 2). Also within

 © 2000 Cancer Research Campaign

**Table 1** Telomere lengths in diagnostic subgroups of malignant lymphomas and in samples from benign lymphoid tissues

| Diagnosis | Number | Telomere length (kbp) | | |
|---|---|---|---|---|
| | | Mean length | | Peak length |
| | | Mean | Range | Range[a] |
| Small cell lymphoma | 22 | 5.1 | 4.0–6.3 | 2.4–>23.0 |
| Large cell lymphoma | 19 | 5.4 | 3.8–6.5 | 2.4–>23.0 |
| Hodgkin's disease | 6 | 5.4 | 4.8–6.0 | 6.2–15.3 |
| Benign lymphoid tissue | 9 | 7.9 | 6.1–9.0 | 6.9–12.6 |

[a] The lymphomas exhibited one or multiple peaks and the peak range values given are the shortest and the longest peaks observed within the subclassified samples. All the benign cases exhibited one broad telomere peak. Due to the presence of lymphoma samples with multiple peaks, mean peak values for the different groups were not calculated. The differences in mean telomere lengths between the lymphoma subgroups and the benign lymphoid tissues were all significant at the 0.01 level.

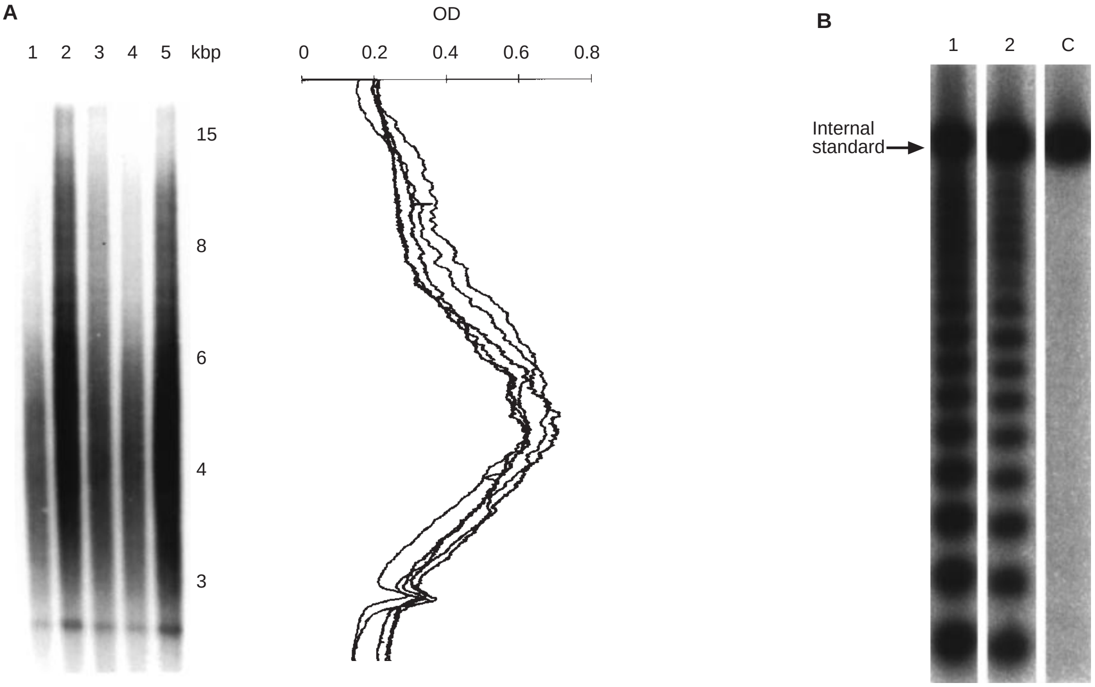

**Figure 1** Patient no. 1 demonstrating remarkably stable telomeres upon relapse and ongoing telomerase activity. (**A**) Patient no. 1 demonstrating unchanged telomere profiles during a period of 57 months in five different samples. Lane 1, diagnostic lymph node sample. Lane 2, lymph node sample 40 months after diagnosis. Lane 3, bone marrow sample 57 months after diagnosis. Lane 4, peripheral blood sample 57 months after diagnosis. Lane 5, pleura effusion sample 57 months after diagnosis. Southern blotting using $(TTAGGG)_4$ probe. Telomere length profiles were obtained by densitometry and were compensated for DNA loading in each individual lane. OD = optical density. (**B**) Patient no. 1 demonstrating telomerase activity at diagnosis and at relapse 40 months later. Lane 1, diagnostic lymph node sample. Lane 2, lymph node sample 40 months after diagnosis. Lane C, negative control (lysis buffer). The level of telomerase activity was analysed at 0.28 μg protein $assay^{-1}$ using the TRAP-assay and the internal standard, ITAS at 15 attograms $assay^{-1}$

the various morphologic subgroups large individual differences were seen. Thus, no obvious differences were seen regarding telomere profile patterns in the lymphoma subgroups at diagnosis.

Concerning samples obtained from the 16 relapsing cases a summary of data regarding the source of tumour material, time from diagnosis, morphologic grading and percentage of tumour cells based on immunophenotyping is given in Table 2. In the relapse group the first sample exhibited peak lengths that ranged from 2.4 to >23 kbp whereas the mean telomere length values showed less variation, from 3.8 to 6.7 kbp, indicating that the cell populations in general harboured chromosomes with a large variation in the length of their telomeres.

In the relapsing lymphomas we decided to determine peak length changes in order to be able to study different populations of cells with unique telomere profiles within a sample, since lymphomas consist of a tumour component as well as normal cells. Studying the peak length changes in the relapsing lymphomas most cases could be divided into samples which exhibited peaks that were stable, decreased or increased in length, even though half of the relapse lymphomas exhibited two peaks while the other half

*© 2000 Cancer Research Campaign* 

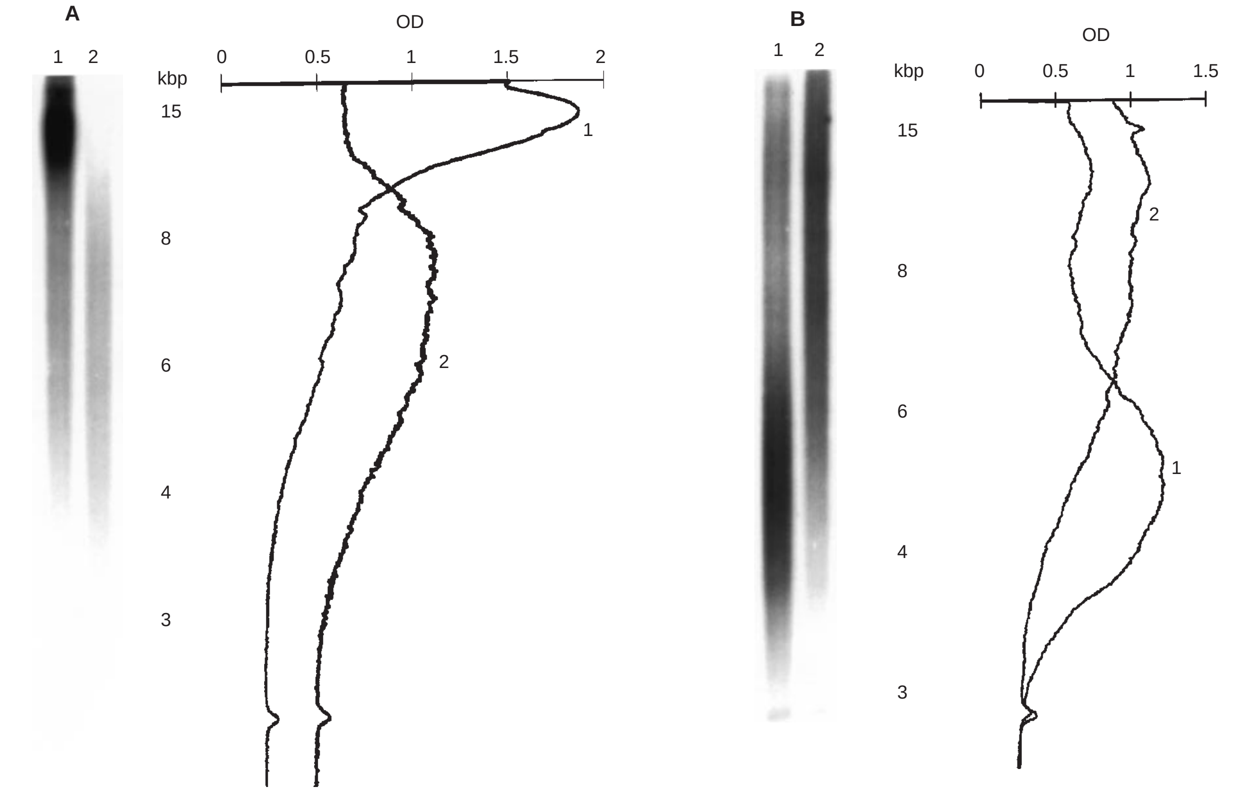

**Figure 2** Compared to the diagnostic samples both decreased and increased telomere lengths were observed in the relapsing tumours. (**A**) Patient no. 14 demonstrating a decrease in telomere length at relapse. Lane 1, diagnostic lymph node sample. Lane 2, lymph node sample 43 months after diagnosis. (**B**) Patient no. 4 showing an increased telomere length at relapse. Lane 1, diagnostic lymph node sample. Lane 2, lymph node sample 47 months after diagnosis. Southern blotting using $(TTAGGG)_4$ probe. Telomere length profiles were obtained by densitometry and were compensated for DNA loading in each individual lane. OD = optical density only exhibited one peak. Deviations of <1 kbp in telomere peak values between different samples were observed in five cases (nos 1, 3, 5, 9, 10; Figure 1A) indicating fairly stable telomere lengths during the course of the disease and six relapsing tumours (nos 6, 7, 12, 14, 15, 16; Figure 2A) demonstrated decreasing peak lengths. Case No. 13 exhibited one stable and very short peak while the longer peak was lost at relapse. Case no. 2 showed increasing telomere peak length while three cases (nos 4, 8, 11; Figure 2B) showed two populations, one with increasing and one with decreasing peak lengths when compared with the respective diagnostic sample. A summary of peak and mean telomere lengths for the relapsing lymphomas are presented in Table 2.

All relapsing lymphomas studied except one demonstrated significant telomerase activity (Table 2). The relative telomerase activity in 0.28 μg protein extract per reaction varied from 0 to 4.88. When the fraction of malignant cells was taken into account the activity was estimated to be from 0 to 12.19, assuming that the normal cells were telomerase negative or had negligible activity.

The relapse data are difficult to describe for the whole group due to the individual character of each case why selected patients are shortly commented below.

*Patient 1* was followed for a total time of 57 months and in five samples taken during this time period a remarkable stability in peak lengths were demonstrated in lymph node, bone marrow, peripheral blood and pleural exudate samples (Table 2 and Figure 1A). Telomerase activity was detected at diagnosis and at relapse after 40 months (Figure 1B).

*Patient 13* showed at both diagnosis and relapse a population of cells with very short and stable telomeres. In the diagnostic sample a second cell population with very long telomeres was present which was lost at relapse. Only in the relapse sample could telomerase activity be detected (Table 2).

*Patient 12* had a lymphoblastic lymphoma showing an obvious reduction in peak lengths in relapse samples from testis and bone marrow (Table 2). The peak length reduction was similar in both compartments.

*Patient 4* was diagnosed with a lymphoma showing two peaks at 10.8 and 4.3 kbp. At relapse the telomere profile was broader with a peak at about 8 kbp (Table 2 and Figure 2B). The fraction of monoclonal B-cells was 40% at relapse compared with 80% at diagnosis, but no peak between 3 and 5 kbp could be revealed indicating a telomere length increase in the population of cells which exhibited the shorter telomere profile.

### DISCUSSION

In the present study the telomere lengths of diagnostic lymphoma samples and benign lymphoid tissue samples were assessed and when comparing the mean telomere lengths of the lymphomas with the benign tissues a significant telomere shortening was

© 2000 Cancer Research Campaign

**Table 2** A summary of relevant data concerning 16 cases with relapsing non-Hodgkin's lymphomas

| Patient | Sample | Source[a] | Time (m)[b] | Diagnosis[c] | Tumour cells (%)[d] | Telomere length (kbp) Peak | Telomere length (kbp) Mean | Telomerase activity[e] | Adjusted telomerase activity[f] |
|---|---|---|---|---|---|---|---|---|---|
| 1 | 1 | LN | 0 | LCBL | 40 | 4.4 | 4.6 | 4.88 | 12.2 |
| | 2 | LN | 40 | LPL | 52 | 4.1 | 4.2 | 0.94 | 1.8 |
| | 3 | BM | 57 | LPL | 64 | 4 | 4.3 | ND | ND |
| | 4 | PB | 57 | LPL | 72 | 4.1 | 4.1 | ND | ND |
| | 5 | PL | 57 | LPL | 71 | 4.2 | 4.3 | ND | ND |
| 2 | 1 | LN | 0 | LCBL | 40 | 4.3 | 4.4 | ND | ND |
| | 2 | PB | 11 | MBC | 36 | 6.8 | 5.1 | ND | ND |
| 3 | 1 | LN | 36 | LCBL | >50 | 5.8 | 5.3 | ND | ND |
| | 2 | LN | 85 | LCBL | ND | 5.8 | 4.9 | 1.73 | ND |
| 4 | 1 | LN | 0 | FCL | >80 | 10.8 + 4.3 | 4.7 | ND | ND |
| | 2 | LN | 47 | FCL | 40 | 8 | 5.6 | ND | ND |
| 5 | 1 | PL | 0 | LCTL | 70 | 13.8 + 7.8 | 5.7 | ND | ND |
| | 2 | PL | 7 | LCTL | 70 | 14.7 + 7.5 | 6.1 | ND | ND |
| 6 | 1 | LN | 0 | LCTL | 65 | 11.1 | 5.9 | 0.14 | 0.21 |
| | 2 | PL | 0 | LCTL | 51 | 10.4 | 6.3 | ND | ND |
| | 3 | PL | 8 | LCTL | 78 | 9 | 5.8 | ND | ND |
| | 4 | PL | 11 | LCTL | 57 | 8.5 | 5 | ND | ND |
| 7 | 1 | Tonsil | 0 | FCL | 40 | >23 + 8 | 5.9 | ND | ND |
| | 2 | LN | 67 | FCL | 60 | 7.5 + 3.3 | 4.5 | 0.32 | 0.53 |
| 8 | 1 | LN | 10 | FCL | 30 | 12.2 | 6.7 | ND | ND |
| | 2 | LN | 68 | FCL | ND | ND | ND | 2.20 | ND |
| | 3 | PL | 89 | FCL | 20 | 15.8 + 9.6 | 5.5 | ND | ND |
| | 4 | LN | 89 | FCL | 80 | 17.3 + 8.4 | 6.2 | 1.61 | 2.01 |
| | 5 | LN | 102 | FCL | 30 | 8.2 | 5.3 | 1.04 | 3.45 |
| 9 | 1 | LN | 0 | LCBL | 60 | 2.4 | 3.8 | ND | ND |
| | 2 | PB | 26 | MBC | >80 | 2.8 | 3.5 | ND | ND |
| 10 | 1 | LN | 0 | MCL | 50 | 6.3 | 5.3 | 0.42 | 0.85 |
| | 2 | Skin | 24 | MCL | 47 | 6.9 | 5.3 | 0.96 | 2.05 |
| 11 | 1 | LN | 0 | LCBL | 50 | 8.2 | 4.8 | 0.93 | 1.85 |
| | 2 | LN | 14 | LCBL | 75 | 8.2 + 2.2 | 4.2 | ND | ND |
| | 3 | LN | 32 | LCBL | 47 | 8.5 + 2.9 | 4 | 0.31 | 0.66 |
| | 4 | LN | 35 | LCBL | 85 | 9.8 + 3 | 4 | 1.91 | 2.25 |
| 12 | 1 | LN | 0 | LCTL | 50 | 11.8 | 6.4 | ND | ND |
| | 2 | Testis | 22 | LCTL | 65 | 7.1 | 5.4 | ND | ND |
| | 3 | BM | 22 | LCTL | 60 | 6.8 | 5.9 | 0.24 | 1.02 |
| 13 | 1 | LN | 0 | FCL | 65 | >23 + 2.4 | 5 | 0 | 0 |
| | 2 | LN | 26 | FCL | 90 | 2.4 | 3.7 | 0.70 | 0.78 |
| 14 | 1 | LN | 0 | LCBL | ND | 10.3 | 5.8 | ND | ND |
| | 2 | LN | 43 | FCL | 40 | 10.8 + 4.9 | 4.7 | 0.64 | 1.60 |
| 15 | 1 | LN | 0 | FCL | 80 | 8.2 + 5 | 5 | ND | ND |
| | 2 | PL | 25 | LCBL | 70 | 6.2 + 2.6 | 4 | ND | ND |
| | 3 | LN | 25 | FCL | 35 | ND | ND | 0.17 | 0.49 |
| 16 | 1 | LN | 0 | FCL | 45 | 14.6 | 6.3 | 0.52 | 1.15 |
| | 2 | LN | 48 | LCBL | 30 | 6.8 | 4.6 | 0.11 | 0.38 |
| | 3 | LN | 73 | FCL | 30 | ND | ND | 0.74 | 2.45 |

[a]IN, lymph node; BM, bone marrow; PB, peripheral blood; PL, pleura fluid. [b]Time in months from diagnosis. [c]LBCL, large B-cell lymphoma; LPL, lymphoplasmacytoid lymphoma; FCL, follicle cell lymphoma; LTCL, large T-cell lymphoma; MCL, mantle cell lymphoma; MBC, monoclonal B-cells detected by flow cytometry. [d]Judged by immunophenotyping and flow cytometry. [e]Relative activity. [f]Telomerase activity value divided by the percentage of tumour cells. ND = not done or inconclusive.

observed in all of the lymphoma subgroups. In addition peak lengths and mean telomere lengths as well as telomerase activity were measured in two to five samples from relapsing non-Hodgkin's lymphomas. The mean telomere length calculation gives a mean value for all telomere ends of all cells in a sample but since lymphoma samples are an admixture between malignant and normal cells the measure is not very informative concerning the telomere lengths of the specific cell populations. Also, since the telomere lengths in the lymphomas were spread over a large kbp range rather large peak value changes gives a small impact on the mean telomere length value. When describing the telomere dynamics of specific cell populations in tissue samples consisting of different cell populations the peak telomere value is the measure of choice. Thus the telomere dynamics of specific cell populations in the 16 relapsing lymphomas were studied concerning the peak lengths. The relapsing lymphomas which all exhibited one or two telomere peaks could be divided into tumours showing stable, decreasing or increasing telomere peak lengths. It was found that all the benign lymphoid tissues exhibited only one broad peak. For the malignant lymphomas the immunophenotyping data were informative because the number of neoplastic cells compared to the number of normal cells corresponded in many cases to the size of the telomere peaks observed. A telomere peak length below 5.0 kbp can be considered to be derived from malignant cells since

*© 2000 Cancer Research Campaign*

not even normal cells from centenarians exhibit such short telomeres (Vaziri et al, 1993; Slagboom et al, 1994). Populations with increasing peak values can also be concluded to be derived from tumour cells since telomere elongation in tumour cells can occur while normal haematopoietic cells exhibit telomere shortening except for normal germinal centre B-cells which are rare in the lymphoma samples (Vaziri et al, 1993; Slagboom et al, 1994; Allsopp et al, 1995; Hiyama et al, 1995*a*; Bryan et al, 1997; Weng et al, 1997). Based on these assumptions it was possible to determine which peaks belonged to normal versus malignant cells in most of the relapsing lymphomas. In the remaining cases we assume that the telomere profiles of the tumour cells resembled those of the normal cells since all these lymphomas except one had a tumour cell fraction of 40% or more and if the tumour cells exhibited a differing telomere profile compared with the normal cells two peaks should have been observed.

Considerable individual differences with respect to the number of peaks as well as peak lengths were found within the diagnostic lymphoma samples, unrelated to morphologic subclassification (peak length, range = 2.4 to > 23 kbp; Table 1). The benign cases by comparison all demonstrated one peak which ranged in length from 6.9 to 12.6 kbp. The difference in age of the patients (12–83 years) from which the benign samples were obtained explains most of the variation in peak length since the telomere length decreases in lymphocytes with age (Vaziri et al, 1993; Slagboom et al, 1994; Allsopp et al, 1995). The large variability seen at diagnosis could be due to malignant transformation in cells with either overall short or long telomeres, which might be a consequence of the number of cell divisions undergone before the transformation event occurred. Thus, one lymphoma might develop from a cell recently recruited from the bone marrow giving a tumour with longer telomeres, whereas another lymphoma evolves from a lymphocyte with a history of many previous cell generations leading to a lymphoma with shorter telomeres.

The telomere dynamics of cells are believed to be influenced by the expression level of telomerase and differing levels of telomerase activity in the diagnostic lymphomas could also explain the telomere length variability seen. We have previously shown that non-Hodgkin’s lymphomas exhibit increased levels of telomerase activity compared to benign lymph nodes (Norrback et al, 1996), a finding further verified in the present study (not shown in figures). The major source of telomerase activity in non-malignant lymphoid tissues is normal germinal centre B-cells which should be low in numbers in the lymphoma samples and thus the activity measured can for the most part be denoted to the tumour component (Norrback et al, 1996). In in vitro manipulation experiments it has been shown that in addition to telomerase activity telomere binding proteins can affect the telomere length of cells but it is not known to what extent tumours exhibit abberant functioning of such proteins (van Steensel and de Lange, 1997; Gravel et al, 1998; LaBranche et al, 1998).

The impact of telomerase activity on the telomere length in our relapsing lymphomas is not obvious from the data presented here, since there are cases with strong telomerase activity which still exhibit significant reduction of the telomere length by time. However, for cases with unchanged or increased telomere lengths ongoing telomerase activity in the tumour cells is one explanation (Figure 1 A,B). Due to the high sensitivity of the TRAP assay (Kim et al, 1994), the activity found is present in a cell fraction of unknown size and there is a theoretical possibility that as little as 0.1–1% telomerase-positive cells can be detected. A strict comparison between telomere length values, which are based on DNA extracted from the whole cell population, and telomerase activity must for this reason be made with caution. However, in the relapsing lymphomas the telomerase activity was of such a magnitude that it is reasonable to believe that a significant fraction of the lymphoma cells de facto had telomerase activity.

One alternative explanation for the telomere length alterations demonstrated in the relapsing lymphomas is that there is a selective loss of cells due to cytostatic therapy or radiation treatment and that the surviving subclones have a certain telomere length constitution which later is manifested in the relapse tumour cell population. This selection of therapy-resistant cells is probably random at the telomere length level. In most of our relapse cases we have performed immunoglobulin gene rearrangement studies and DNA fingerprinting analysis, showing that the original clone is preserved but that clonal changes not seldom occur by time which can support the idea of selective loss and repopulation (Rosenquist, 1998). The diagnostic samples from patients 7 and 13 both contained a potential ALT population which exhibited a peak length beyond 23 kbp and cells with shorter telomeres (Table 2). It is interesting to note that upon relapse the potential ALT cells were lost while the cells with the shorter telomere profiles prevailed.

In the future we can foresee the development of telomerase inhibitors for use in the treatment of malignant diseases. Anti-telomerase drugs are expected to be the most effective if they are used to treat tumours with short telomeres and are administered after the tumour burden has been reduced using conventional therapy. Theoretically tumours can be divided into two main groups according to their expected suitability as targets for anti-telomerase treatment. The first group represents tumours which have been derived from a telomerase-negative cell and would be expected to frequently reactivate telomerase late in tumorigenesis after the senescence barrier has been inactivated. Inhibition of telomerase in these tumours would have a cytotoxic effect on the tumour cells. The other group of tumours are either derived from stem cells or other telomerase competent cells and would be expected to retain telomerase expression throughout tumorigenesis. These tumours would be expected to much more frequently possess an intact senescence programme and exhibit longer telomeres compared to the tumours in the first group. Telomerase inhibition in these tumours would have a delayed cytostatic effect.

Lymphomas belong to the second group of tumours since normal lymphocytes express telomerase upon cell cycle progression whereas it is possible that many solid tumours belong to the first group described and thus would be more suitable targets for anti-telomerase therapy. In the present study however, six out of 16 malignant lymphomas exhibited decreased telomere peak lengths upon relapse and only four of the relapsed tumours showed peak lengths below 6.0 kbp. This indicates that only a fraction of these tumours would have been suitable targets for telomerase inhibition treatment. It should be noted that we cannot exclude the possibility that a few of the relapsing lymphomas significantly elongated their telomeres through the action of telomerase and that the use of telomerase inhibitors could have prevented the relapse. Due to these findings however, the telomere length of relapsing tumours after conventional therapy is an important field of investigation. Such studies will ultimately determine the extent at which anti-telomerase drugs will become successful therapeutic regimens in the clinical setting.

© 2000 Cancer Research Campaign

### ACKNOWLEDGEMENTS

This study was supported by grants from The Swedish Cancer Society, The Medical Faculty, Umeå University and Lion's Cancer Research Foundation, Umeå University.

### REFERENCES

Allsopp RC, Chang E, Kashefi-Aazam M, Rogaev EI, Piatyszek MA, Shay JW and Harley CB (1995) Telomere shortening is associated with cell division in vitro and in vivo. *Exp Cell Res* **220:** 194–200

Bodnar AG, Ouellette M, Frolkis M, Holt SE, Chiu C-P, Morin GB, Harley CB, Shay JW, Lichtsteiner S and Wright WE (1998) Extension of life-span by introduction of telomerase into normal human cells. *Science* **279**: 349–352

Bryan TM and Reddel RR (1997) Telomere dynamics and telomerase activity in in vitro immortalised human cells. *Eur J Cancer* **33**: 767–773

Bryan TM, Englezou A, Dalla-Pozza L, Dunham MA and Reddel RR (1997) Evidence for an alternative mechanism for maintaining telomere length in human tumours and tumour-derived cell lines. *Nat Med* **3**: 1271–1274

Feng J, Funk WD, Wang S-S, Weinrich SL, Avilion AA, Chiu C-P, Adams RR, Chang E, Allsopp RC, Yu J, Le S, West MD, Harley CB, Andrews WH, Greide CW and Villeponteau B (1995) The RNA component of human telomerase. *Science* **269**: 1236–1241

Gravel S, Larriveé M, Labrecque P and Wellinger RJ (1998) Yeast Ku as a regulator of chromosomal DNA end structure. *Science* **280**: 741–744

Harris NL, Jaffe ES, Stein H, Banks PM, Chan JKC, Cleary ML, Delsol G, De Wolf-Peeters C, Falini B, Gatter KC, Grogan TM, Isaacson PG, Knowles DM, Mason DY, Muller-Hermelink H-K, Pileri SA, Piris MA, Ralfkiaer E and Warnke RA (1994) A revised European–American classification of lymphoid neoplasms: a proposal from the international lymphoma study group. *Blood* **84**: 1361–1392

Hiyama E, Yokoyhama T, Hiyama K, Yamakido M, Santo T, Kodama T, Ichikawa T and Matsuura Y (1995*a*) Alteration of telomeric repeat length in adult and childhood solid neoplasias. *Int J Oncol* **6**: 13–16

Hiyama K, Hirai Y, Kyoizumi S, Akiyama M, Hiyama E, Piatyszek MA, Shay JW, Ishioka S and Yamakido M (1995*b*) Activation of telomerase in human lymphocytes and hematopoietic progenitor cells. *J Immunol* **155**: 3711–3715

Holt SE, Norton JC, Wright WE and Shay JW (1996) Comparison of the telomeric repeat amplification protocol (TRAP) to the new TRAP-eze telomerase detection kit. *Methods Cell Sci* **18**: 237–248

Härle-Bachor C and Boukamp P (1996) Telomerase activity in the regenerative basal layer of the epidermis in human skin and in immortal and carcinoma-derived skin keratinocytes. *Proc Natl Acad Sci USA* **93**: 6476–6481

Jean-Louis M and Claude H (1998) G-quadruplex DNA: a target for drug design. *Nat Med* **4**: 1366–1367

Kim NW, Piatyszek MA, Prowse KR, Harley CB, West MD, Ho PLC, Coviello GM, Wright WE, Weinrich SL and Shay JW (1994) Specific association of human telomerase activity with immortal cells and cancer. *Science* **266**: 2011–2015

LaBranche H, Dupuis S, Ben-David Y, Bani M-R, Wellinger RJ and Chabot B (1998) Telomere elongation by hnRNP A1 and a derivative that interacts with telomeric repeats and telomerase. *Nat Gen* **19**: 199–202

Levy MZ, Allsopp RC, Futcher AB, Greider CW and Harley CB (1992) Telomere end-replication problem and cell aging. *J Mol Biol* **225**: 951–960

Mehle C, Mieczyslaw AP, Ljungberg B, Shay JW and Roos G (1996) Telomerase activity in human renal cell carcinoma. *Oncogene* **13**: 161–166

Meyerson M, Counter CM, Eaton EN, Ellisen LW, Steiner P, Caddle SD, Ziaugra L, Beijersbergen RL, Davidoff MJ, Liu Q, Bacchetti S, Haber DA and Weinberg RA (1997) hEST2, the putative human telomerase catalytic subunit gene, is up-regulated in tumour cells and during immortalization. *Cell* **90**: 785–795

Morales CP, Holt SE, Ouellette M, Kaur KJ, Yan Y, Wilson KS, White MA, Wright WE and Shay JW (1999) Absence of cancer-associated changes in human fibroblasts immortalized with telomerase. *Nat Genet* **21**: 115–118

Morin GB (1989) The human telomere terminal transferase enzyme is a ribonucleoprotein that synthesizes TTAGGG repeats. *Cell* **59**: 521–529

Norrback K-F and Roos G (1997) Telomeres and telomerase in normal and malignant haematopoietic cells. *Eur J Cancer* **33**: 774–780

Norrback K-F, Dahlenborg K, Carlsson R and Roos G (1996) Telomerase activation in normal B lymphocytes and non-Hodgkin's lymphomas. *Blood* **88**: 222–229

Norrback K-F, Enblad G, Erlanson M, Sundström C and Roos G (1998) Telomerase activity in Hodgkin's disease. *Blood* **92**: 567–573

Rosenquist R (1998) The immunoglobulin heavy chain gene as a clonal marker in lymphoma and leukemia. Umeå University medical dissertations, new series No 558-ISSN 0346–6612

Slagboom PE, Droog S and Boomsma DI (1994) Genetic determination of telomere size: a twin study of three age groups. *Am J Hum Genet* **55**: 876–882

Strahl C and Blackburn EH (1996) Effects of reverse transcriptase inhibitors on telomere length and telomerase activity in two immortalized human cell lines. *Mol Cell Biol* **16**: 53–65

van Steensel B and de Lange T (1997) Control of telomere length by the human telomeric protein TRF 1. *Nature* **385**: 740–743

Vaziri H, Schächter F, Uchida I, Wei L, Zhu X, Effros R, Cohen D and Harley CB (1993) Loss of telomeric DNA during aging of normal and trisomy 21 human lymphocytes. *Am J Hum Genet* **52**: 661–667

Vaziri H, Dragowska W, Allsopp RC, Thomas TE, Harley CB and Lansdorp PM (1994) Evidence for a mitotic clock in human hematopoietic stem cells: loss of telomeric DNA with age. *Proc Natl Acad Sci USA* **91**: 9857–9860

Weng N-P, Levine BL, June CH and Hodes RJ (1995) Human naive and memory T lymphocytes differ in telomeric length and replicative potential. *Proc Natl Acad Sci USA* **92**: 11091–11094

Weng N-P, Granger L and Hodes RJ (1997) Telomere lengthening and telomerase activation during human B cell differentiation. *Proc Natl Acad Sci USA* **94(20)**: 10827–10832

Wright WE, Shay JW and Piatyszek MA (1995) Modifications of a telomeric repeat amplification protocol (TRAP) result in increased reliability, linearity and sensitivity. *Nucleic Acids Res* **23**: 3794–3795

Wynford-Thomas D (1997) Proliferative lifespan checkpoints: cell-type specificity and influence on tumour biology. *Eur J Cancer* **33**: 716–726

*© 2000 Cancer Research Campaign*
